# Forgotten, excluded or included? Students with disabilities: A case study at the University of Mauritius

**DOI:** 10.4102/ajod.v6i0.359

**Published:** 2017-08-29

**Authors:** Sameerchand Pudaruth, Rajendra P. Gunputh, Upasana G. Singh

**Affiliations:** 1 Faculty of Ocean Studies, University of Mauritius, Mauritius; 2 Department of Law, Faculty of Law and Management, University of Mauritius, Mauritius; 3 College of Law and Management Studies, School of Management, Information Technology and Governance, Discipline of Information Systems and Technology, University of KwaZulu-Natal, South Africa

## Abstract

**Background:**

Students with disabilities in the tertiary education sector are more than a just a phenomenon, they are a reality. In general, little attention is devoted to their needs despite the fact that they need more care and attention.

**Objectives:**

This paper, through a case study at the University of Mauritius, sought to answer some pertinent questions regarding students with disabilities. Does the University of Mauritius have sufficient facilities to support these students? Are students aware of existing facilities? What additional structures need to be put in place so that students with any form of disability are neither victimised, nor their education undermined? Are there any local laws about students with disabilities in higher education?

**Method:**

To answer these questions and others, an online questionnaire was sent to 500 students and the responses were then analysed and discussed. The response rate was 24.4% which showed that students were not reticent to participate in this study.

**Results:**

Our survey revealed that most students were not aware of existing facilities and were often neglected in terms of supporting structures and resources. ICT facilities were found to be the best support that is provided at the University of Mauritius. The right legal framework for tertiary education was also missing.

**Conclusion:**

Ideally, students with disabilities should have access to special facilities to facilitate their learning experiences at tertiary institutions. Awareness about existing facilities must also be raised in order to offer equal opportunities to them and to enable a seamless inclusion.

## Introduction

According to the 2011 population census, there are 59 868 people with disabilities in the Republic of Mauritius, which corresponds to a disability prevalence rate of about 5% (Statistics Mauritius 2015). Although this is lower than the global disability prevalence rate (WHO 2011), the Republic of Mauritius has been implementing laws and policies for the well-being of people with disabilities since 1976 with the promulgation of the *National Pensions Act* 1976 (Supreme Court 2017). The Republic of Mauritius signed the United Nations Convention on the Rights of Persons with Disabilities (UNCRPD) in 2007 and ratified it in 2010 although certain reservations were made in relation to Articles 9.2(d), 9.2(e), 11 and 24.2(b) (UNTC 2017). Article 24 of the UNCRPD stipulates that all member states shall ensure that people with disabilities should be given equal opportunities to follow tertiary education (UNCRPD 2006). Article 28 of the United Nations Convention on the Rights of the Child also guarantees the right to a free primary education (OHCHR 2016). According to the World Disability report (WHO 2011), there are about 1 billion people in this world who have some form of disability. For example, in Malawi, more than 97% of children with disabilities do not have access to education (Chilemba 2013). Despite the fact that many countries, including Mauritius, have signed and ratified the UNCRPD, most of them, have failed in their obligations to conceptualise and implement the right to education for students with disabilities as envisaged by the international conceptual approaches and legal standards of inclusive standard in the UNCRPD. The UNCRPD defines a disability as a long-term physical, mental, intellectual or sensory impairment, which may hinder a person’s full and effective participation in society on an equal basis with others. Persons with disabilities are often the subject of severe social stigma, discrimination and harassment which consequently force them to live under the misguided belief that their lives are shameful and not worthy of respect (Peltzer 2014; Waterstone & Stein 2008). This has resulted in the creation of a culture of marginalisation and routine discrimination, in a society which is already on the downfall, especially in developing countries where children with disabilities do not have access to education. Poverty, unemployment and social tensions (civil wars, discrimination) also account for this. Therefore, there is an uncontested connection between poverty and disability (Degener 2017; Rubey 2002; Schneider, Mokomane & Graham 2016).

Because of political or social reasons, some countries are even more affected than others, in terms of equal rights and equal opportunities, with adverse affects education. The UNCRPD has played an important role in the improvement of the dignity of all persons with disabilities, but there are many challenges ahead in terms of its implementation at the national level. Some legislations such as the *Mental Health Care Act* 1998 (Act 24/1998), the *HIV/AIDS Act* 2006 (Act 31/206) and *Mauritius Mental Health Association Act* 1974 (Act 8/19740) are indirectly involved in the protection of persons with disabilities, to allow these citizens to enjoy their rights, on an equal footing with others. The *Training and Employment of Disabled Persons Act* 1996, the *National Council for Life Rehabilitation of Disabled Persons Act* 1986, the *Society for the Welfare of the Deaf Act* 1968, the *Lois Lagesse Trust Fund Act* 1983, the *Child Protection Act* 1994, the *Ombudsperson for Children Act* 2003 and the *Unemployment Hardship Relief Act* 1983 are some of the legislations which have been promulgated to help, protect and promote a more inclusive life for disabled persons in Mauritius. All the above-mentioned legislations are available from the Supreme Court of the Republic of Mauritius (Supreme Court 2017).

The primary aim of this research is to report on students’ perceptions regarding support structures available to students with disabilities in the hope that this will encourage states and tertiary education institutions to implement policies and measures to support the education of students with disabilities, based on the findings of this study. This research aims at increasing the awareness about the facilities that exist in tertiary institutions for students with disabilities at the University of Mauritius. Through this study, the researchers hope to ensure that these students are not overlooked in tertiary education, and also make a recommendation for the adoption of e-learning as a possible solution to integrate students with disabilities into the mainstream tertiary education. In the same vein, this paper attempts to encourage tertiary institutions to provide facilities and support structures to enable students with disabilities to enjoy the same benefits on campus as mainstream students, thus providing them equal rights and opportunities for education. After an introduction, the structure of this paper follows a traditional pattern. A brief review of related literature is discussed in the next section, before the research methodology adopted in this study is described. The findings of this study are then presented. This empirical paper ends with a conclusion and a brief presentation of the limitations of this study. Finally, the list of cited references is provided.

## Literature review

Georgeson, Mamas and Swain (2015) define a disabled student as ‘any student who has a sensory, cognitive, physical or psychological impairment’. The White Paper on Education (in South Africa) defines a person with a disability as ‘a person limited or impaired in one or more functional activities which prevents full and equal academic, social and economic participation’ (UKZN 2004). The impairment may be permanent, recurring or transitory and may be sensory, physical, cognitive or psychological (UKZN 2004). There is a general increase in the number of students with disabilities entering higher education in developed countries (AHEAD 2015; Altbach, Reisberg & Rumbley 2009; NCES 2016). Especially when distance education institutions understood the special needs of students with disabilities, they began to develop support systems that assisted these students by providing materials using alternative forms of media, and by adopting enabling technologies (Kirkup 2015).

The Open University of Japan (OUJ) provides support for students with disabilities by allowing them to study from home. This includes adopting special arrangements for examinations and classroom-based activities, extensive use of radio and TV for lecture transmission; providing transcripts and captions for all learning materials and making texts available in digital form for screen reader users (Cooper 2015).

However, despite this increase in e-learning facilities, students with disabilities continue to display lower retention rates when compared to mainstream students (Lichiello 2012; NCLD 2014). This lower retention rate may be because of institutional barriers. Georgeson et al. (2015) outline that disabled university students face many challenges, which may include a lack of suitable facilities, struggles to access learning materials, a lack of understanding and respect from both fellow students and academics, to name a few. In a study in the United Kingdom, it was found that the issues faced by students with disabilities were exacerbated by the lack of a coordinated effort, across the institution, to address these special needs (Cooper 2015).

Furthermore, locally, in Africa, Van Jaarsveldt and Ndeya-Ndereya (2015) identified technological barriers, a lack of awareness and poor liaison among the institutional stakeholders and lecturers who distanced themselves from the responsibility of providing learner support to students with disabilities as the challenges that students with disabilities faced. Research has shown that students with disabilities feel they have to work harder than other students, because they have to manage a double workload – their disability and their studies (Gorman 1999).

Uncaptioned videos, disorganised websites and course materials that cannot be read by screen readers, or accessed without a mouse, and educators who have little knowledge of how to ensure that their courses are accessible compound the difficulties faced by students with disabilities (Cooper 2015). The introduction of relevant technologies can provide support to them in their learning. Often it is thought that technology can help to reduce the barriers to equitable education for students with disabilities and thus promote better integration of students with disabilities into mainstream higher education (Ahmad 2015; DFID 2015). Data collected from students with disabilities in a UK higher education institution showed that students with disabilities lack the correct digital capital to enable them to succeed within higher education environments. Thus, it is important for higher education institutions to ‘conceptualise’ and ‘organise’ technology-related support services for students with disabilities, to support and promote access to equitable educational experiences and outcomes (Georgeson et al. 2015).

The adoption of digital technologies, such as virtual learning environments and e-learning, can help to achieve increased levels of accessibility and inclusion (Douce 2015). However, simply adopting these technologies does not ensure ‘accessibility’. Educators or administrators have to make sure that these relatively new digital tools are indeed accessible to all. The solution maybe lies in using standard design principles which are universally accepted. When creating e-learning materials for students with disabilities, learning materials must cater for all four major disability categories: visual, hearing, motor and cognitive impairments. The emphasis of universal design is on social inclusion while accessibility focuses on the implementation of specific features and processes. Thus, this inclusive nature of universal design and accessibility can greatly enhance the interaction of students with disabilities with e-learning (Van Rooij & Zirkle 2016). It is essential that the developers of the e-learning environment, aiming to integrate accessibility into the e-learning environment, familiarise themselves with both the capabilities and limitations of the institution’s technology, early in the development process, to minimise the repetitive nature of having to re-design elements that cannot be supported by the current infrastructure.

Adopting a learner-centred approach may require academics to adapt their educational practices to enhance learning for all students, to include disabled students. The design and implementation of e-learning spaces and learning materials that are accessible to all students can be accomplished by providing multiple means of representation, action and expression, as well as engagement, which meet recognised design principles. Successful e-learning environments that support students with disabilities are flexible and robust enough to afford opportunities to students with disabilities to enter inclusive education settings, without being marginalised (Van Jaarsveldt & Ndeya-Ndereya 2015). Ultimately, technology provides unique opportunities to assist students with disabilities to learn more easily. The key to achieve this though is to develop e-learning environments that make their learning processes interactive, accessible and inclusive (Yahya et al. 2015).

The transition from paper-based to digital and eventually to web-based learning has brought with it new uses of technology to support students with disabilities, as well as challenges for these students (Kirkup 2015). Although e-learning enhances the availability of access to content material, the full potential of this benefit can only be realised if the student has access to the appropriate assistive technologies (AT). As Georgeson et al. (2015) explain, students with disabilities obtain more meaningful learning experiences if they have access to AT like alternative interfaces (e.g. screen readers), reading tools (e.g. text-to-speech), recording tools (e.g. voice recording), writing tools (e.g. word prediction), planning tools (e.g. mind-mapping software) and communication tools (e.g. synthetic speech). Inaccessible design of e-learning portals can be a barrier to students with disabilities. Thus, technology is a ‘double-edged sword’ and if students with disabilities are exposed to poorly designed e-learning environments in higher education, they will be on the ‘wrong side of a second digital divide’. To improve the adoption of e-learning, academics are urged to improve their practices and support for the use of AT. This also includes empowering students with disabilities to be more active users of technology and assisting them in making more informed decisions about the benefits of actively engaging with technology to enhance their learning processes.

Furthermore, to improve the experiences of students with disabilities in higher education institutions, it is imperative that we listen to their voices, which provide us with information on the challenges they face while being learners at these institutions. The aspect of sharing good practices should not be neglected either, as case studies represent a powerful approach to help to learn from academics’ experiences, thus increasing ‘the volume of important voices’ (Douce 2015). As Cooper (2015) states, there is a need for ‘formal research that documents the experiences of academics with disability students in online learning’. More strategies need to be explored to address the complex issues of access and inclusion. The creation of an inclusive learning environment at higher education institutions will remain elusive if academics distance themselves from providing learner support to students with disabilities. Merely transferring the responsibilities to support services, such as the unit for students with disabilities, at the institution will frustrate the learning of these students (Van Jaarsveldt & Ndeya-Ndereya 2015).

In Japan, the United Kingdom and the United States, the law is seen very much as a driver for change (Cooper 2015), forcing higher education institutions to implement facilities to support students with disabilities. However, it must be stressed that abiding by the law alone will not affect a dramatic change. A greater participation of all stakeholders in higher education is required to ensure that students with disabilities become a part of the mainstream students in higher education institutions. As Van Jaarsveldt and Ndeya-Ndereya (2015) have stated, ‘beyond legislation and institutional policies relating to students with disabilities, academics should accept responsibility for and have an understanding of accessibility and the establishment of inclusive learning environments’. It is thus imperative to expand our research to identify methods, which can empower students with disabilities, and provide them with deeper and more enriched learning experiences.

In South Africa, inclusive education was implemented following the introduction of the *Higher Education Act* of 1997 (Council on Higher Education 1997) and the National Plan for Higher Education in 2001 (Ministry of Education 2001). The Education White Paper 6 on Special Needs Education (2001) outlines the inclusion of students with disabilities in broad terms and specifically mentions that higher education institutions are required to draft their own institutional plans to support students with disabilities (Department of Education 2001). These institutional plans must include the strategies and steps that will be taken by the institution to implement the guidelines provided by the legislation. More recently, the Department of Higher Education and Training in South Africa approved the White Paper for Post-School Education and Training which aims to produce a single, coordinated post-school education and training system (DHET 2013).

Many South African universities have established specialised disability units, to facilitate and coordinate specific support services for students with disabilities (UKZN 2004). These services may include sign language interpretation, Braille services, infrastructure, equipment and software installation, to facilitate easier learning (Van Jaarsveldt & Ndeya-Ndereya 2015). These units have also created institutional policies to help support their processes. These institutional policies aim to provide current and prospective students and staff who have disabilities with the opportunity for full participation at the universities of their choice. For these policies to be successfully implemented, they should be reviewed on a regular basis in consultation with people with disabilities to allow them to make input into improvements required to make their opportunity for full participation in university life possible (Hall & Healey 2004).

Despite the reservations made in the UNCRPD, the Republic of Mauritius has a number of domestic laws for the protection and inclusion of people with disabilities or special needs in mainstream life. In 2008, the *Equal Opportunities Act* was enacted to prevent all kinds of discrimination based on disability (Supreme Court 2017). The *Building Control Act* 2013 ensures that all new public buildings and infrastructures are accessible to people with disabilities (Supreme Court 2017). The *Training and Employment of Disabled Persons Act* requires that all employers having 35 or more staff should employ at least 3% people with disabilities. A number of public bodies such as the Disability Empowerment Unit, the National Council for the Rehabilitation of Disabled Persons and the Training and Employment of Disabled Person Board have been set up to carry out government policies based on domestic laws and the recommendation of the UNCRPD. People with disabilities who are victims of discrimination can seek redress directly not only from the police or the office of public prosecutions but also from the Equal Opportunity Tribunal, National Human Rights Commission, the Office of the Ombudsman, the Office of the Attorney General and the committee on Economic, Social and Cultural Rights (MSSNSRI 2016; Statistics Mauritius 2015). Depending on the severity of their disability and their age, people with disabilities also receive a pension every month. Thus, we can see that in general, there exist sufficient laws in the Republic of Mauritius, which have been put in place for the well-being of people with disabilities.

Although the Republic of Mauritius currently does not have any statutes specifically for the education of people with disabilities, it has been doing a lot for promoting inclusive education despite the UNCRPD reservation made on Article 24.2(b). The Special Education Needs and Inclusive Education (SEN & IE) Policy and Strategy (MoEHR 2006) states that:

‘An inclusive educational system, starting in the early years of development, and aimed at responding to the educational needs of each student through a child-centred pedagogical approach and a flexible and adapted curriculum will help each child of Mauritius develop his/her full potential.’ (p. 5)

Currently, in Mauritius, many students with disabilities attend specialised schools but the government is working towards the goal of total inclusion (MoEHR 2006). Students with disabilities are offered free transport by the government. Laws and regulations have been amended to make the school environments more accessible. Many nongovernmental ogranisations like the Global Rainbow Foundation (GRF 2016) help students with disabilities in the acquisition of assistive devices for walking, hearing, talking, sight, etc., so that they can be included in mainstream education. In terms of tertiary institutions, to our knowledge, out of 10 publicly funded institutions and 53 private ones (TEC 2017), only the University of Mauritius has so far produced formal regulations with regard to the facilities that are available to students with disabilities (University of Mauritius 2016). The other universities, colleges and schools provide tailored support on a case-by-case basis. In the past few years, the Ministry of Education (MoEHRTESR 2016) has been giving scholarships to students with disabilities who wish to study at a local public university.

## Research design and methodology

An online self-administered or self-completion questionnaire (Appendix 1), which included a mixture of open-ended and closed questions, was used for the data collection process. This questionnaire was benchmarked against similar studies undertaken internationally (Dent et al. 2015; Healey et al. 2015; Meyer et al. 2012; Roualdes 2013). The primary data collected were derived from the anonymous responses received to the online questionnaire. The questionnaire focused on gathering information on the general awareness of students regarding students with disabilities, the perceived views of students about the support structures available to students with disabilities, the awareness of laws by students to support students with disabilities, the obstacles students with any form of disability are facing in tertiary and higher education and the role of e-learning in providing a more supportive learning environment for students with disabilities.

The population for this study was students at the University of Mauritius. The questionnaire was electronically distributed through the use of Google Forms to 500 students, which included those with disabilities. The sample selected adopted a combination of convenience and purposive sampling. Convenience, as the students were available for participation at the university, and purposive, because it focused solely on the University of Mauritius. No exclusion criteria were adopted in this process. Post-data collection, the quantitative responses were analysed using simple data analysis techniques, while the qualitative data were extracted into themes for discussion. Responses are reported with an R, indicating a respondent in the discussion of the findings.

Therefore, this article attempts to reflect a better understanding of this phenomenon, and thus enable more effective measures against it, in the Mauritian context, to provide better facilities and encourage students with disabilities to adapt and succeed in tertiary education.

### Ethical considerations

Ethical clearance procedures were completed. Participants were informed of the voluntary nature of the survey, the anonymity of their responses and the purpose of the survey.

## Findings and results

As presented earlier, the questionnaire was distributed to 500 students, which represents about 4% of the total student population of the University of Mauritius. A total of 122 responses, which represents a response rate of 24.4%, were received in total, with 6 responses from students with disabilities.

### Overview of students with disabilities

**FIGURE 1:**
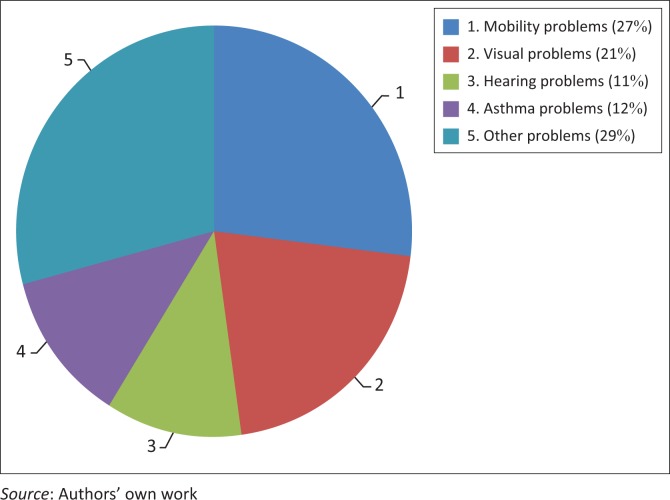
Types of disabilities among students of the University of Mauritius.

Figure 1 represents an overview of the students with disabilities in this study. For the 2015–2016 academic years, there were 52 students (0.4% of the student population) who declared that they have some form of disability or an other. About 27% of these 52 students have mobility problems (hand, leg or other parts of the body), about 21% have visual problems, 11% have hearing problems, another 12% have asthma problems and the rest have other medical problems such as diabetes, stammering, difficulty to concentrate and other rare or complex conditions. This information was received from the University of Mauritius.

**FIGURE 2:**
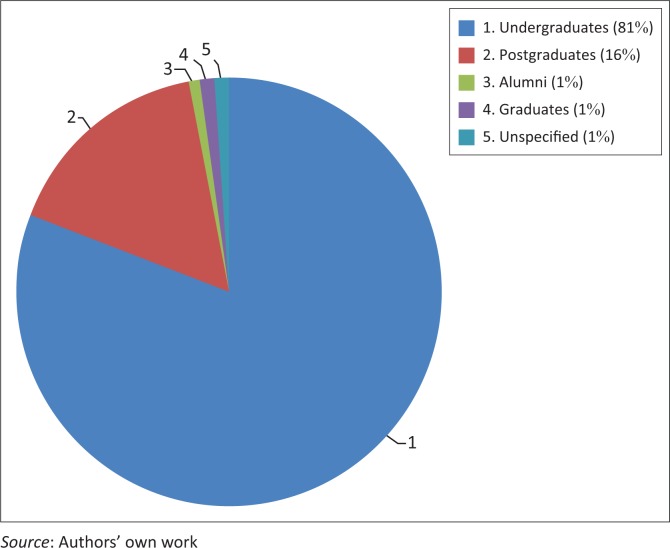
Level of study of the participants.

The questionnaire was distributed in such a way so as to get students from different levels of study at the University of Mauritius. Figure 2 shows the demographics of participants including undergraduates (81.1%), postgraduates (16.4%), alumni (0.01%), graduates (0.01%) and unspecified (0.01%). Thus, the overwhelming majority of students were undergraduates while a few who responded to the invitations were no longer students of the university. Out of the 122 respondents, 54 (44.3%) participants were male students and 68 (55.7%) were female students.

**FIGURE 3:**
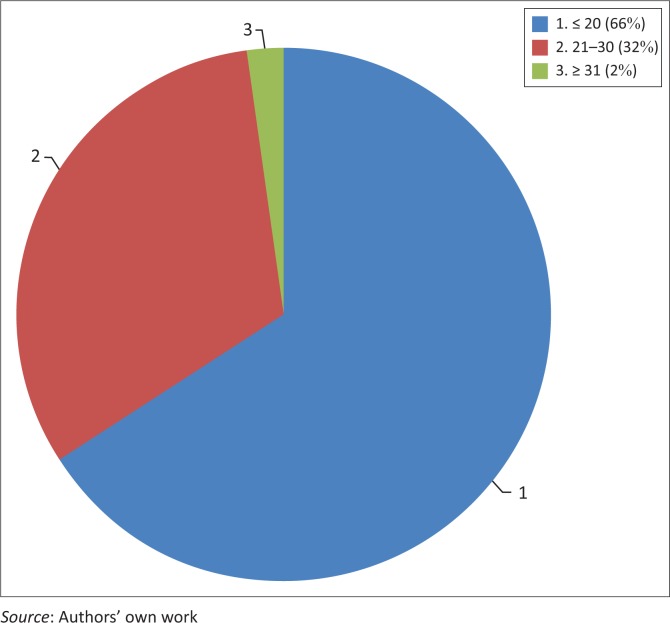
Age distribution of participants.

Figure 3 represents the age distribution of the participants. Most participants were younger students. Participants who were 20 and under totalled 81 (66.4%), 39 (32%) were between 21 and 30 years, while just 2 (1.6%) were older learners who were between 31 and 45 years of age. There were 99 (81.1%) participants who were full-time students, 12 were part-timers (9.8%), 9 (0.07%) participants identified themselves as both full-time and part-time students and 2 (0.02%) were distance learning students.

### Awareness levels and support structures

Following the demographic data collection, the next part of the questionnaire gathered information about the respondents’ awareness levels of students with disabilities. It also gathered their views on the current support structures available for students with disabilities. Nineteen (15.6%) students indicated that they have a close relative or friend who had or has some form of disability, which impacted on their ability to study at a tertiary institution. Furthermore, 81 (66.4%) respondents affirmed that they are aware that there are students with disabilities at the university.

Despite being aware of the fact that there are students with disabilities at the University of Mauritius, 80 respondents (65.6%) mentioned that they were unaware of the amount of funds that are injected by the University of Mauritius in order to provide the necessary facilities and support structures for students with disabilities. One hundred and three (84.4%) respondents stated that they felt public and private tertiary institutions in Mauritius are ill-equipped to provide these facilities.

**FIGURE 4:**
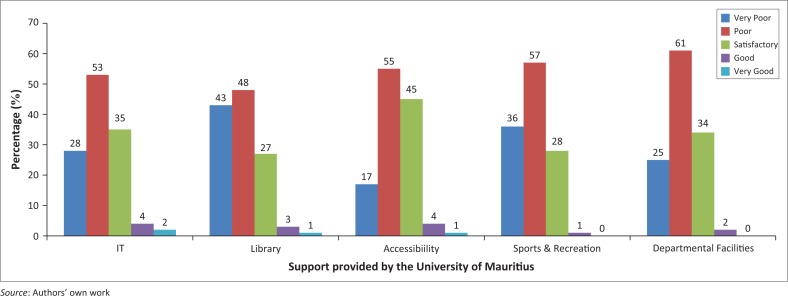
Provision for students with disabilities.

Figure 4 summarises the respondents’ views on the support provided by the University of Mauritius for students with disabilities, in terms of IT, library facilities, general accessibility, sports and recreational facilities and departmental facilities. All facilities were consistently rated by the majority of the respondents as either ‘poor’ or ‘very poor’. General accessibility was ranked the highest with 45 (36.9%) respondents rating these facilities as satisfactory. Although specific IT facilities were not investigated, general IT support 6 (4.9%) topped the ‘good to very good’ category.

In many higher education institutions abroad, facilities to support students with disabilities include: rooms that are designed to support students with mobility disabilities, specialist software for students with sight and dyslexic disabilities, alternate access facilities to buildings and support group structures for students with disabilities (Georgeson et al. 2015; Lichiello 2012). At the University of Mauritius, some of these facilities have been provided. For example, lectures for students with mobility problems are done in places which are easily accessible to them. Students with visual problems are allowed 25% additional time to submit their scripts. The font size on question papers is also enlarged. Colours may also be used as appropriate. There is also provision for Braille-printed exam papers. For students with other types of disabilities, the faculties should provide necessary and appropriate facilities as far as is possible with the resources available and without compromising academic standards.

The adoption of e-learning, and its associated tools, was viewed favourably by most respondents (92.6%) as facilities that could enhance and encourage better learning for students with disabilities. The greatest motivations for these opinions was flexibility: ‘the ability to allow them the flexibility to work from home’ – respondents R6, R18, R20. This was supported by respondents R8 and R14, who said that these students ‘will not have to travel long distances’ (R8, R14) which is sometimes difficult for those who do not have transport facilities. However, respondent R9 felt that e-learning would be inappropriate as these students ‘want to attend university’ like regular students do. Respondent R5 also supported this view stating that ‘e-learning would make them feel like they are different from other students and hence deprive them from social interaction which is in a way rejecting them from society’.

The questionnaire thereafter moved on to investigating awareness and appropriateness of laws associated with protecting and assisting students with disabilities. A large number of respondents, totalling 71 (58.2%), were not aware of any disability laws in Mauritius. Of the 51 (41.2%) respondents who were aware of disability laws in Mauritius, 12 respondents (23.5%) stated that these laws do not protect students with disabilities, while 6 respondents (11.8%) were unsure. The motivations provided by these respondents supported the statistics above, as most of the comments discussed laws that protect disabled citizens in general, rather than specifically focusing on students with disabilities. Respondent R7 stated that laws for people with disabilities ‘gives them the opportunity to get jobs … they do not suffer from discrimination’ (R7, male, student). This was supported by respondent R87 who indicated that there are ‘… laws that are enforced for recruitment of disabled persons’ (R87, female, student) and respondents R113 and R121 who spoke about ‘incentives and protection against discrimination’ (R113, R121, male, student). Respondent R49 stated that ‘these laws are not well enforced in every institution … they should be reviewed and amended to help the student’ (R49, female, student). Only four respondents (3.3%) were aware of any foreign laws that supported people with some form of disabilities.

### Feedback from students with disabilities

The next part of the survey focused on views from students with disabilities. Only six responses (4.9%) were received from students with disabilities. Nevertheless, their feedback is of crucial importance for this study. The largest form of disability indicated was visual impairment (three respondents) followed by mobility difficulty (one respondent), partial hearing loss (one respondent) and one asthmatic student.

Five of these respondents (83.3%) indicated that their parents provided them with the necessary medical support to assist them in being an active member of society. All six participants (100%) further affirmed that they are unaware of any university disability advisor at the University of Mauritius who they can contact to discuss their requirements and problems.

**FIGURE 5:**
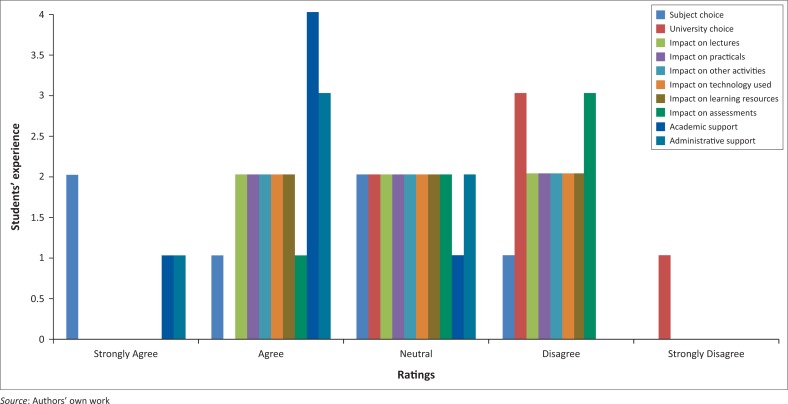
Views of students with disabilities about their university experience.

The next set of questions gathered the views of students with disabilities about their actual experience at the University of Mauritius, which are presented in Figure 5.

Three (50%) respondents indicated that that their choice of subjects was affected by the limitations they faced with their disability. The selection of university at which to study did not seem to have a role to play. Only two respondents (33.3%) faced difficulties with lectures, tutorials, practicals, technical facilities and learning resources. One (16.7%) respondent indicated that his disability was a barrier for him to complete his assessments successfully. The respondents’ experience with regard to the support they obtained from their lecturers was very positive, as the majority of them (83.3%) were very satisfied with their lecturers’ role in their studies. Support staff was equally helpful, as indicated by 4 (66.7%) respondents.

In the general comments section, respondents R13, R20 and R25 were keen on learning about the laws in Mauritius that specifically catered for students with disabilities. An interesting suggestion was made by R9 with regard to increasing the accessibility of the university campus by students with disabilities. The respondent proposed that by allowing students with disabilities from different faculties to suggest inaccessible places (which are important to them), the University of Mauritius will be in a better position to take appropriate measures to make the campus more accessible for such students.

## Discussion of findings

Of the 500 students who were sent the questionnaire, only 122 responded, out of which 6 were students with disabilities. Compared to the percentage of students with disabilities at the University of Mauritius, this is a relatively high response rate. The University of Mauritius has a population of 12 500 students. This could indicate that these students were not reticent to participate in this study to demonstrate their knowledge or voice their concerns. For ethical and data privacy reasons, it was not possible to get a named list of students with disabilities from the University of Mauritius. It was noted that participants were aware of family members and fellow students with disabilities.

The University of Mauritius may not be doing enough to inform the student community about the support that they offer for students with disabilities. This was indicated by the large number of respondents who were not aware of the facilities and support structures that are provided to students with disabilities. The results indicate that the IT facilities are the best support provided at the university for both able-bodied students and students with disabilities. This correlates with the large support for the adoption of e-learning as a tool to promote better interactivity and inclusivity of students with disabilities into the mainstream education structure.

The awareness of laws supporting people with disabilities was restricted to laws relating to social justice and higher education. There is a general feeling among the respondents that laws associated with better support and inclusion of students with disabilities into tertiary education are required, or if such laws exist, they should be made aware of them. There was very little knowledge of foreign laws associated with students with disabilities. Responses received from students with disabilities, although small in number, provided insights into their experiences while studying at the University of Mauritius. Although they are provided with support from their parents, they seem to be getting little support at the institution itself. Lecturers and support staff were identified as being sensitive to the disabilities of students, but a formal support structure at the University of Mauritius seems to be lacking. This calls for greater investigation by the relevant stakeholders, at the university, to find ways to enhance the learning experience at the institution, for students with disabilities.

## Conclusion

This study indicates that the University of Mauritius has been making some effort to provide special facilities and arrangements to support students with disabilities. Nevertheless, some support structures, such as student liaison officers, appropriate recreational and sport facilities and special access features have been identified as lacking at the university by the students. The introduction of these facilities coupled with increased awareness of them will ensure that students with disabilities are able to pursue their studies with the same amount of effort as those without any disabilities. Most students identified e-learning as having a key role to play in the support of students with disabilities. Essentially, the problems and barriers that are faced by students with disabilities in tertiary education must not be overlooked, in order to offer equal opportunities to every student. To conclude, students with disabilities are neither forgotten nor excluded at the University of Mauritius; however, more effort is certainly required for their seamless inclusion.

## Limitations

Two limitations associated with this study are the small number of participants with disabilities and that the context is restricted to the University of Mauritius. Future research should look at expanding this to include the participation of more students with disabilities and more tertiary institutions in Mauritius and abroad in order to get a more holistic picture of the situation.

## Appendix 1

**All information would be kept private and confidential.**

**Please complete this survey anonymously. Do not write your name, address or any personal details anywhere in the form.**

**Researchers:** Mr S Pudaruth, Dr RP Gunputh and Dr UG Singh

***A survey on general awareness of laws and facilities for students with disabilities at the University of Mauritius***

1. **What level of study are you in?**
  - Undergraduate
  - Postgraduate
  - Alumni
  - Other
2. **Gender**
  - Male
  - Female
3. **At what age did you commence your studies?**
  - 20 and under
  - 21–30
  - 31–45
  - Over 60
4. **How do you study?**
  - Full-time
  - Part-time
  - Other
5. **Do you have any close relatives or friends who suffer from any form of disability which consequently has a serious impact on his/her studies in a tertiary education?**
  - Yes
  - No
6. **Are you aware that there are students at your University who suffer from any form of disability? ***
  - Yes
  - No
7. **How much funds do you think the tertiary education where you are registered actually is ready to finance so that students who are suffering from any form of disability may be provided with the necessary facilities they need?**
  - More than 1 million rupees annually
  - More than 2 million rupees annually
  - More than 3 million rupees annually
  - More than 4 million rupees annually
  - I have no idea
8. **Do you think that public and private tertiary institutions are well equipped to provide facilities for students suffering from disabilities?**
  - Yes
  - No
    ***Please rate Questions 9 to 13 using the scale provided.***
    Very Poor Poor Satisfactory Good Very Good
9. **How would you rate the University’s IT provisions for disabled students?**
10. **How would you rate the University’s Library provision for disabled students?**
11. **How would you rate the accessibility, for disabled student, of the University’s campus, in general?**
12. **How would you rate the University’s Sports and Recreation facilities, for disabled students?**
13. **How would you rate the facilities in your Department/School to support disability requirements?**
14. **Do you think e-learning would encourage students who are suffering from any form of disabilities to register on e-learning programmes to be graduated?**
  - Yes
  - No
15. **Motivate your answer to Question 14**
16. **Are you aware of any disability laws in Mauritius?**
  - Yes
  - No
17. **Do you think this/these law(s) protect(s) people suffering from disabilities?**
  - Yes
  - No
  - Other:
18. **Why? Motivate your answer to Question 17**
19. **Do you suffer from any disability?**
  - Yes
  - No
20. **Which of the following categories best describes your disability? (Please select as many as apply to you)**
  - Dyslexia
  - Unseen disability (e.g. asthma, epilepsy)
  - Deaf/Hearing impairment
  - Wheelchair user/Mobility difficulty
  - Asperger’s syndrome/Autism
  - Mental health difficulty
  - Blind
  - Eyesight problems
  - Other:
21. **Do your parents provide the necessary medical support (medical treatment/medical devices) in order for you to cope properly with your studies at the university you have joined and registered? ***
  - Yes
  - No
22. **Are you aware of any University Disability Advisor at the University? ***
  - Yes
  - No
    ***Please rate the statements in questions 23 to 32 using the rate scale provided* ***
    Strongly Agree Agree Neutral Disagree Strongly Disagree
23. **My disability affected my choice of subject(s) to study.**
24. **I chose to study at this University because it offers support for disability students.**
25. **I have faced disability-related barriers, at the University, which have impacted on my learning experience in lectures.**
26. **I have faced barriers, related to my disability, at the University, which have impacted on my learning experience in on-campus laboratory and/or practical work.**
27. **I have faced barriers, related to my disability, at the University, which have impacted on my learning experience in other on-campus classes (e.g. seminars, tutorials).**
28. **I have faced barriers, related to my disability, at the University, which have had an impact on my use of technical facilities (e.g. computers, multimedia, audio/visual equipment, photocopying).**
29. **I have faced barriers, related to my disability, at the University, which have affected my use of learning resources, (e.g. lecture handouts, computer-assisted learning packages).**
30. **I have faced barriers, related to my disability, at the University, which have affected my experience with assessments/tests/examinations.**
31. **Academic staff (e.g. lecturers/tutors) have been supportive and helpful when I have approached them with concerns about to disability-related barriers I have experienced**
32. **Support staff (e.g. administrators, technicians, librarians) have been supportive and helpful when I have approached them with concerns about disability-related barriers I have experienced**
33. **Are you aware of any foreign legislation which may improve our local legislation on disability law, with particular focus on tertiary institutions?**
34. **Do you have any suggestions/recommendations to improve this survey?**
